# Acute Appendicitis as a First Presentation of Unimetastatic Gastric Adenocarcinoma

**DOI:** 10.7759/cureus.57051

**Published:** 2024-03-27

**Authors:** Leila Laghmiche, Salome Salmon, Sebastien Michiels

**Affiliations:** 1 Surgery, Université Libre de Bruxelles, Brussels, BEL

**Keywords:** unimetastatic, appendix metastasis, gastric adenocarcinoma, gastric cancer, appendicitis

## Abstract

Appendicitis is one of the most common causes of abdominal surgery emergencies worldwide. Here, we report a case of acute appendicitis as a primary presentation of gastric adenocarcinoma with appendiceal metastasis and no evidence of other lesions. This case can be added to only a few other reported cases, showing a similar situation that can help clarify the spread of gastric adenocarcinoma.

## Introduction

Appendicitis is the most common cause of emergency abdominal surgery worldwide. Malignant tumors of the appendix are rare, representing 1% of all large intestine tumors. In more than 50% of malignant tumors of the appendix, acute appendicitis is the first presentation [1].

Gastric cancer is the fifth most common cancer and the third most deadly. Its main risk factors include *Helicobacter pylori* infection, a high-salt diet, and a low-fiber diet. The treatment of gastric cancer primarily relies on surgical resection, although (neo)adjuvant chemotherapy improves survival [2]. Its metastasis is commonly localized in the liver, peritoneum, ovaries, and lungs [3]. The first description of gastric cancer metastasizing to the appendix was given in 1951 by Goldfarb and Zuckner [4].

We present the case of a patient with appendicitis as the first presentation of gastric adenocarcinoma metastasis.

## Case presentation

**Figure 1:**
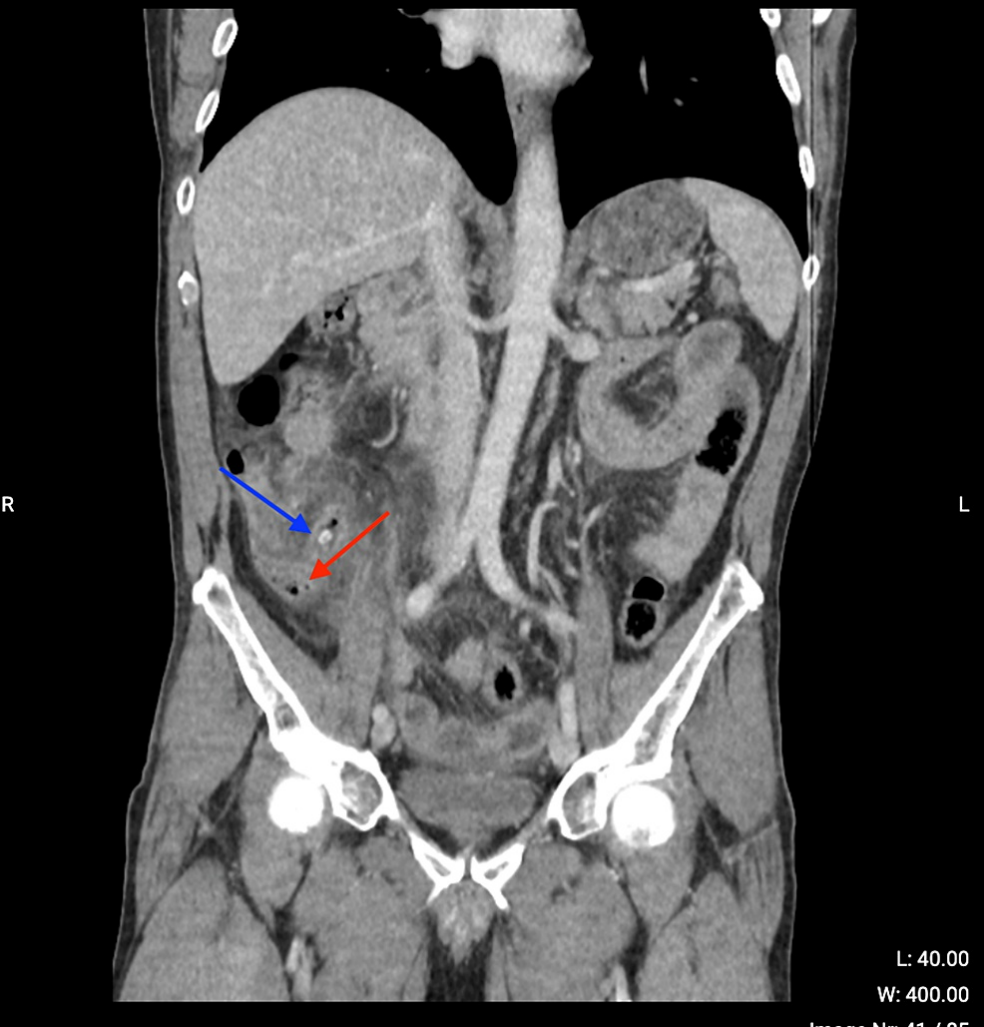
Abdominal CT scan. CT scan showing air alongside the appendix (red arrow) and stercolith (blue arrow).

**Figure 2:**
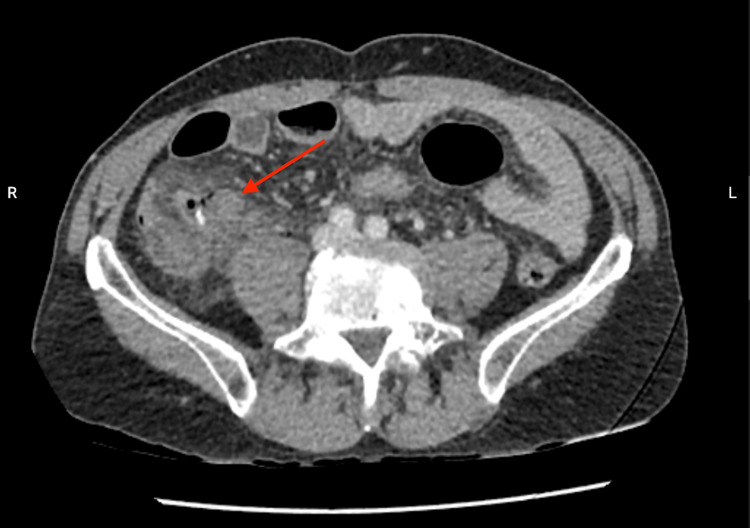
Abdominal CT scan. CT scan showing fat infiltration around the appendix (red arrow).

**Figure 3:**
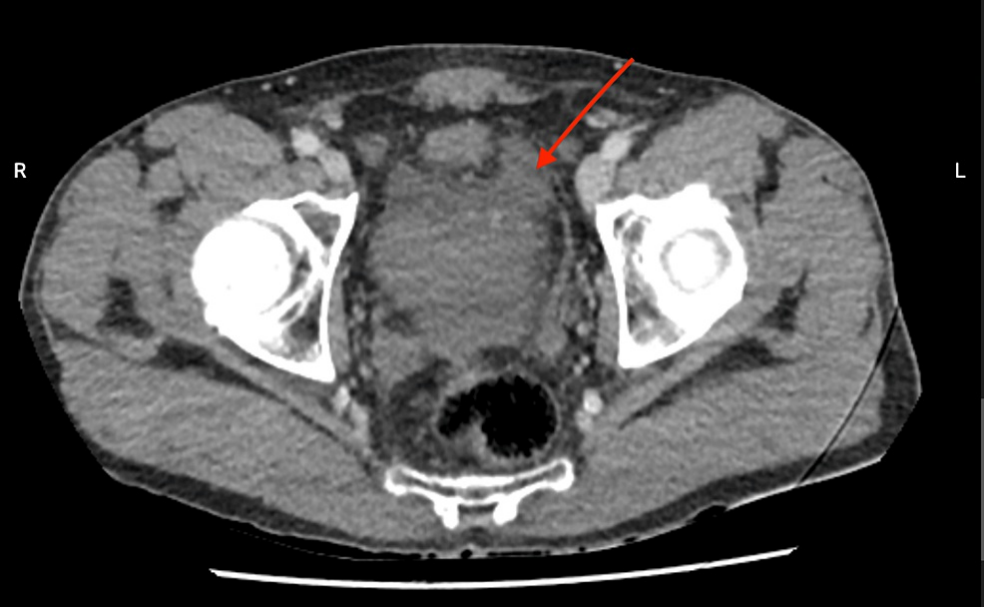
Abdominal CT scan. CT scan showing liquid in the peritoneal space in the pelvis (red arrow).

A 66-year-old male presented to the emergency room complaining of continuous abdominal pain for 24 hours, nausea without pyrexia, and episodes of vomiting and diarrhea. An abdominal CT scan with contrast showed a stercolith in the appendix, distension and thickening of the appendix, and air alongside the appendix (Figure 1). It also revealed peri-appendicular fat infiltration (Figure 2) and a small quantity of liquid in the pelvic space (Figure 3).

Blood tests revealed hyperleukocytosis (18,030/µL) with a predominance of neutrophils (14,510/µL) and an inflammatory syndrome with a C-reactive protein level of 94.84 mg/L. A laparoscopic appendicectomy was performed and showed a necrotic appendix with peritonitis and purulent liquid in the abdominal cavity. Due to the inflamed and friable appendix, the laparoscopy was quickly converted to a laparotomy with a Jalaguier incision. Ultimately, due to the fragility of the walls adjacent to the appendix and a perforation located at its base, an ileocecectomy with a primary anastomosis was performed. After the abdominal cavity was flushed and drained, no other lesions were found.

During postoperative follow-up, the patient received intravenous amoxicillin-clavulanic acid antibiotherapy and was placed on a low-residue diet (consisting of rice, toast, pasta, and steamed potatoes) for two days and then realimented without complications. The culture of the intra-abdominal fluid collected during the surgical procedure yielded a positive result for *Enterococcus faecium* and *Escherichia coli* resistant to ampicillin. The antibiotherapy was adapted and switched to intravenous ciprofloxacin-metronidazole and tigecycline.

Anatomopathology showed a moderately differentiated adenocarcinoma in the small intestine, colonic wall, and appendix. The immunohistochemistry (anti-CK7 and anti-CK20) was positive which suggested an upper digestive cancer origin and the presence of vascular tumor embolization. The surgical margins were negative, and all the lymph nodes were free from tumoral invasion.

The patient underwent an extensive assessment through imaging. This assessment included a gastroscopy and echo-endoscopy with biopsies, an abdominal MRI, and a positron emission tomography-computed tomography (PET-CT) scan.

**Figure 4:**
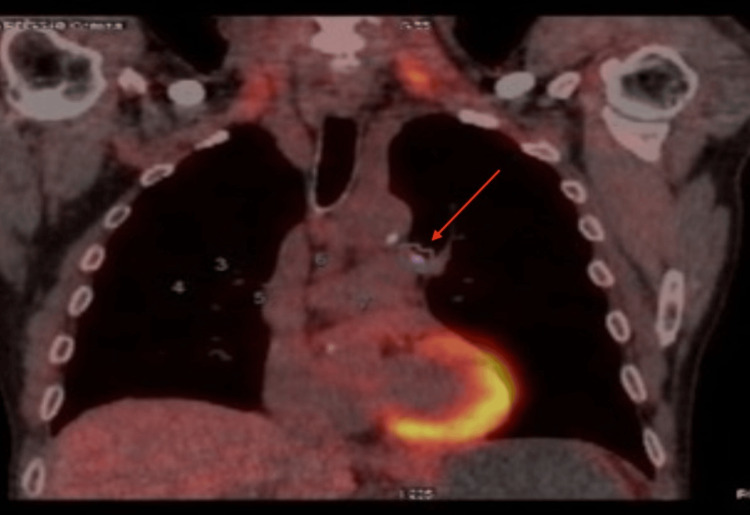
Positron emission tomography-computed tomography scan showing an inflammatory mediastinal adenopathy.

**Figure 5:**
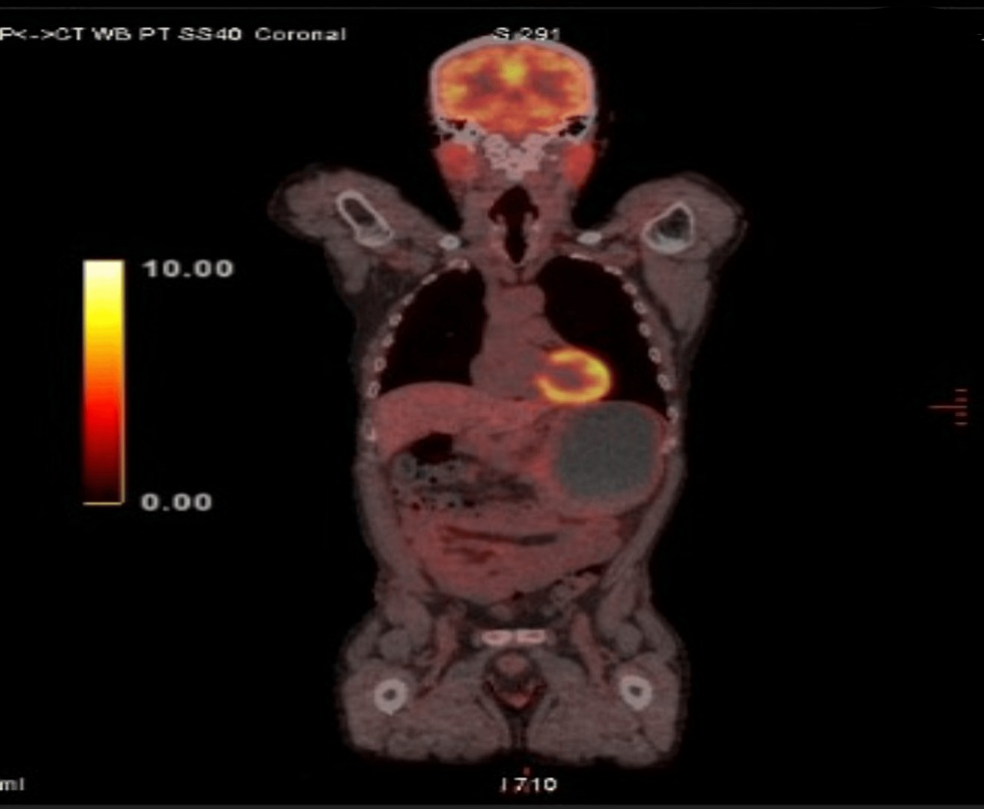
Positron emission tomography-computed tomography scan showing a complete regression of the adenopathy. This image was obtained three months after the previous scan shown in Figure 4.

The gastroscopy revealed a voluminous gastric tumor (echoendoscopic stratification UT3N1). Biopsies displayed a low-grade gastric adenocarcinoma infiltrating focally and no lymphovascular invasion. Abdominal MRI showed no signs of dissemination, and a PET-CT scan showed pulmonary hilar adenopathy that was likely inflammatory (Figure 4). Considering the lymphadenopathies identified in the initial PET scan with suspicion of an inflammatory pathology, a follow-up conducted three months later revealed a complete regression of the adenopathy (Figure 5).

The patient underwent placement of a port-a-cath (PAC) as well as seven cycles of neoadjuvant chemotherapy using the FOLFOX (folinic acid, fluorouracil, and oxaliplatin) regimen.

Five months after the initial diagnosis, imaging surveillance indicated localized disease without distant metastatic extensions.

An exploratory laparoscopy done to establish the Sugarbaker score revealed one adhesion on the surgical site measuring <0.5 cm and the gastric tumor was palpated but not visible, which resulted in a Peritoneal Cancer Index score of 1.

Considering the favorable response to neoadjuvant chemotherapy and the stability of the disease, the patient underwent a distal gastrectomy with hyperthermic intraperitoneal chemotherapy (HIPEC). Postoperative follow-up proceeded uneventfully. Subsequently, the patient was monitored through imaging surveillance (thoracoabdominal CT) and blood tests every three months.

## Discussion

Gastric cancer metastasizing to the appendix was first described in 1951 by Goldfarb and Zuckner [4], and a few such cases have been reported since [5-11].

Although the mechanism of gastric adenocarcinoma dissemination remains unclear, peritoneal dissemination is a possible hypothesis. It is also possible that this was a case of peritoneal single metastasis in the appendix.

The spread of gastric cancer may differ depending on the cancer type and localization (cardia or non-cardia) in the stomach. While cancer localized in the cardia seems to spread to the liver, lungs, and bones (extraperitoneal), non-cardia cancer seems to spread through the peritoneum (e.g., to the ovaries) [12].

In the present case, anatomopathology showed a vascular embolus which supports the hypothesis of vascular dissemination. However, vascular dissemination is unlikely because no metastasis was found in the liver, through which the gastric veinous drainage operates. In the case presented here, gastric cancer was “unimetastatic,” which is quite uncommon.

An oligometastatic cancer is described as a cancer presenting with only a small number of metastases in one or two parts of the body. In our case, the patient exhibited only one metastasis in a single part of the body from a typically aggressive cancer. This can potentially represent a subclassification of oligometastatic tumors, although it was not mentioned in the literature review by Guckenberger et al. [13].

Oligometastatic gastric cancer has been recognized as a clinical entity that would benefit from different management and treatment, involving initial chemotherapy with reevaluation of response. If the response is complete or partial with the persistence of an oligometastatic presentation, surgical resection of the primary lesion and metastases with adjuvant chemotherapy is indicated. However, the prognosis remains poor [14]. In the case presented here, the patient was treated with an ileo-cecectomy first and a gastrectomy with HIPEC, both with healthy margins. Three-year follow-up showed no relapse.

## Conclusions

This case report adds to the current understanding of the metastatic dissemination of gastric cancer. Future studies on the spread of gastric cancer should examine differences in the spread of classic metastatic and oligometastatic cancers, noting that oligometastatic cancer is likely more aggressive than unimetastatic cancer as we presented. The limited dissemination will probably have an impact on both prognosis and treatment. In this case, the patient had a good prognosis despite the tumor’s aggressiveness.
